# Food insecurity and associated factors among households with under-5 children in slum communities in Ibadan, Nigeria

**DOI:** 10.1186/s12889-023-17051-2

**Published:** 2023-11-02

**Authors:** Tinuola Maria Oderinde, Olayinka Stephen Ilesanmi, Aanuoluwapo Adeyimika Afolabi

**Affiliations:** 1 Department of Community Medicine, University College Hospital, Ibadan, Oyo State Nigeria; 2 Africa Centre for Disease Control, Addis Ababa, Ethiopia; 3 Technical Services Directorate, MSI Nigeria Reproductive Choices, Abuja, Nigeria; 4 Plus-Circle Community Health Advancement Organization, Ado Ekiti, Nigeria

**Keywords:** Food security, Household food insecurity, Under-5 children, Slums, Nigeria

## Abstract

**Introduction:**

Food insecurity is a leading cause of childhood morbidity and mortality. This study assessed the prevalence of household food insecurity and its associated factors among under-5 children in Ibadan, Nigeria.

**Methods:**

This was a cross-sectional household survey of 1,027 under-5 children and their caregivers in urban and rural slums in Ibadan. We used an electronic interviewer-administered, semi-structured questionnaire adapted from the Nigeria Demographic Health Survey and Household Food Insecurity Access Scale was used to report sociodemo-economic characteristics, food insecurity, and anthropometric measurement. The household food insecurity scale consisted of nine questions graded from 0 (Never) to 3 (Often) computed to determine the presence of food insecurity. Nutrition indices were computed, and the results were classified according to World Health Organization 2006 cut-off points. Chi-square tests were used to assess associations between food insecurity and the independent variables. Binary logistic regression analyses were conducted to identify the predictors of food insecurity (α = 0.05).

**Results:**

The mean ages of the caregivers and under-5 children were 31.7 ± 7.47 years and 34.49 ± 15.8 months respectively. Overall, 530 (51.7%) children were females, and 765 (74.5%) had normal weight for height. In all, 195 (19.0%) households had food insecurity, while 832 (81.0%) households had food security (Chi-square = 103.364, p = < 0.001). Under-5 children living in urban slums were seven times more likely to experience household food insecurity compared to those in rural slums (AOR = 6.859, 95%CI = 4.524–10.509, p = < 0.001).

**Discussion:**

Household food insecurity was more prevalent in urban slums. Strengthening of the school health program would help identify children with nutritional deficits, and improve the overall health status of children living in slum communities.

## Background

Food insecurity remains a critical global issue affecting millions of individuals, particularly vulnerable populations such as under-5 children [1]. Being food secure is a basic human right, yet owing to various social, economic, and environmental disparities, more than 850 million people face food insecurity, approximately 12% of the total world’s population [2, 3]. Developing countries in North America, Asia and Africa are intensely affected. In Africa, Nigeria is listed among countries grappling with food insecurity [3].

According to the World Food Programme, more than 16 million people in Nigeria are food-insecure. The Northeastern region of Nigeria accounts for approximately half of the prevalence of food insecure persons in the country [4]. Food insecurity is a leading cause of morbidity and mortality, especially among vulnerable populations such as children, women and people living with HIV infection [5–7]. It is associated with a reduction in the quality of life, a higher proportion of cardiovascular diseases including hypertension and heart disease, metabolic disorders, depression, and poor health outcomes across several illnesses during childhood and adult life [8–11]. It is also strongly related to household income level though not all households living in poverty are food insecure. Food insecurity is a major problem faced by slum dwellers [12].

Understanding the prevalence and predictors of food insecurity among households with under-5 children in slum communities is the first step towards designing effective interventions to address this global challenge. This study therefore aimed to assess the prevalence of food insecurity and its associated factors among households with under-5 children in slum communities in Ibadan, Nigeria.

## Methods

### Study area

The study was conducted in Ibadan, the capital of Oyo State located in the Southwestern part of Nigeria. At present, the slums in Ibadan are situated in six urban and rural local government areas (LGAs) namely Akinyele, Ibadan North, Ibadan North-East, Ibadan North-West, Ibadan South-West, and Ibadan South-East [12]. There are at least 10 slum communities in Ibadan, and Ibadan North LGA has the largest number of slums. This study was conducted in five slum communities: three rural and two urban slums in Ibadan North Local Government Area, Oyo State. The LGA has a total projected population of more than 600,000 based on the 2006 census [13].

### Study design and population

This was an analytic cross-sectional study. The study population comprised under-5 children and their respective caregivers in households located within urban and rural slums. For this study, a household was defined as ‘a group of persons with common provision of food, shelter and other essentials for living’ [14]. The informants were the caregivers of the under-five children. From households that had under five children, one was selected by simple random sampling and underwent anthropometric measurement.

For this study also, a selected community was identified and labelled as an “urban slum” if it is situated in an urban area colony that lacked basic amenities such as durable housing, sufficient living area for inhabitants, access to improved water and sanitation facilities.’ A selected community was labelled “rural slum” if it was situated in the least developed part of a rural area. We included households with an under-five child whose caregiver could answer questions about the child, and others about the purchase and preparation of meals, and dispensing food budget for the household.

### Sample size calculation

The sample size was calculated using the formula for cross-sectional studies, with a standard normal deviate corresponding to 5% level of significance (α) = 1.96, prevalence of food insecurity among slum residents in Ibadan (81%) [15], a design effect/correction factor of 1.5, and a non-response rate of 15%. This corresponded to a sample size of 417 households.

### Sampling technique

The World Health Organization (WHO) modified cluster sampling technique [16] was used to select houses in the slum areas. A cluster was defined as five houses existing side by side. More than 750 clusters were present, out of which we selected 350. A starting point was picked in each of the slums by the spin of a bottle. This point corresponded to a central location in each slum. All the eligible caregivers, beginning from the selected starting point, were interviewed. From the first house, interviewers moved clockwise in an alternate fashion to select the next house or compound. The process continued till we attained the sample size in each of the communities.

In all selected households where an under-five child lived, that child’s anthropometric measurements were taken. Where there was more than one under five child in a household, only one child was selected by balloting.

### Study instruments

The study utilized an interviewer-administered, semi-structured questionnaireadapted from the Nigeria Demographic Health Survey (NDHS) and Household Food Insecurity Access (HFIA) Scale for Measurement of Food Access developed by The Food and Nutrition Technical Assistance and USAID [16].

The questionnaire had four sections, namely sociodemo-economic characteristics, household food insecurity, 24-hr dietary recall, and anthropometric measurement. The questionnaire was translated into Yoruba and Hausas versions and back-translated to English to ensure it retained the original meaning. The questionnaire was then loaded onto the KoBoToolBox application that was downloaded onto android phones used by the research assistants for data collection. 

### Data collection

The questionnaire was pretested at Ojoo, in Akinyele LGA; another community with urban and rural slums. Ten percent of the sample size was used to calculate the number of questionnaires used for pre-test. Data were analyzed and findings from the pre-test were used to address any identified ambiguity in the questionnaire. The Cronbach alpha reliability coefficient was calculated (α = 0.95) to assess the internal consistency of the different aspects of the questionnaire, after which appropriate modifications were made to produce the final questionnaire for the study. Seven research assistants were employed and trained over two days on the study objectives, eligibility criteria, data administration using the KoBoToolBox application, ethics of research, protocol for anthropometry measurement, and COVID-19 preventive measures. All research assistants were at least bi-lingual for English and Yoruba and/or Hausa language. Data collection lasted two months.

.

### Data analysis

Data were downloaded from the KoboToolBox server in Microsoft Excel, cleaned, and then exported to and analyzed using SPSS version 25. Appropriate recoding was done. Categorical variables were summarized using frequencies and proportions. Quantitative variables were summarized using mean and standard deviation. 

Chi-square tests were used to assess the association between the primary outcome variable (food insecurity) and the independent variables. Binary logistic regression analyses were conducted to identify the predictors of food insecurity. Variables that were significant at 10% level of statistical significance in the first logistic regression model were pooled into the second model. Adjusted odds ratio and 95% confidence interval were reported.

The HFIA scale consisted of nine (9) questions with a focus on participants’ experiences with food scarcity, the associated inconvenience, and their behavioral responses to food insecurity [16]. Each of these questions inquired whether a particular condition of food insecurity was experienced in a household (yes/no), as well as its frequency of occurrence (rarely/sometimes/often). Response scores ranged from a minimum of zero to a maximum of 27. These questions served to assign households along a continuum of severity. Hence, each of the nine questions was given a score based on the response. Any question where the answer was “no” was scored 0. Where the response was “rarely”, “sometimes” or “often”, the question was scored 1, 2 or 3 respectively.

Then, the households were placed in their proper HFIA category using the key below:

HFIA category = 1 if [(Q1a = 0 or Q1a = 1) and Q2 = 0 and Q3 = 0 and Q4 = 0 and Q5 = 0 and Q6 = 0 and Q7 = 0 and Q8 = 0 and Q9 = 0]

HFIA category = 2 if [(Q1a = 2 or Q1a = 3 or Q2a = 1 or Q2a = 2 or Q2a = 3 or Q3a = 1 or Q4a = 1) and Q5 = 0 and Q6 = 0 and Q7 = 0 and Q8 = 0 and Q9 = 0]

HFIA category = 3 if [(Q3a = 2 or Q3a = 3 or Q4a = 2 or Q4a = 3 or Q5a = 1 or Q5a = 2 or Q6a = 1 or Q6a = 2) and Q7 = 0 and Q8 = 0 and Q9 = 0]

HFIA category = 4 if [Q5a = 3 or Q6a = 3 or Q7a = 1 or Q7a = 2 or Q7a = 3 or Q8a = 1 or Q8a = 2 or Q8a = 3 or Q9a = 1 or Q9a = 2 or Q9a = 3].

The categories were 1 for Food secure, 2 for Mildly food insecure, 3 for Moderately food insecure and 4 for Severely food insecure [16]. Finally, dichotomous categories comprising of food secure and insecure households were created from the above groups. Specially, the three categories: mildly food insecure, moderately food insecure and severely food insecure were combined into a new group (food insecure).

Nutrition indices were computed using the WHO Anthro 3.2.2 and the results were classified according to WHO 2006 cut-off points [17]. The length/height-for-age Z-score, weight-for-age Z-score and weight for length/height Z-score of children were calculated. The nutritional status of under-five children, were defined by using the WHO growth standards [18]. Children with height-for-age Z-scores between − 2 standard deviation (SD) and − 3 SD were ‘stunted’, while those with height-for-age Z-scores below − 3SD were categorized as having ‘severe stunting’. Children with weight-for-age Z-scores below − 2SD were ‘underweight’. Those with weight-for-height Z-scores were between − 2SD to − 3SD were ‘wasted’, those with scores below − 3SD were categorized as having ‘severe wasting’; those with weight-for-height Z-scores above + 1SD were grouped as ‘overweight’ while those with scores above + 2SD were grouped as ‘obese’. Children with weight-for-age Z-scores between − 2SD to + 2SD were considered as ‘normal’, while those with weight-for-height Z-scores between − 2SD and + 1SD were considered ‘normal’.

Socio-economic status was computed using the information on the ownership of house items such as stove, electric fan, refrigerator etc. Wealth quintiles were then generated by calculating the wealth distribution cut-off points. The wealth quintiles were Q1 = first (Poorest), Q2 = second (Poorer), Q3 = third (Average), Q4 = fourth (Richer), Q5 = fifth (Richest), while “Q1” denoted “lowest wealth index” and “Q5”, “the highest wealth quintile.

## Results

The mean age of the caregivers was 31.7 ± 7.47 years. Overall, 463 (45.1%) had ages between 28 and 35 years, and 895 (79.6%) were employed. The mean age of the children was 34.49 ± 15.8 months, and 530 (51.7%) were females. Overall, 765 (74.5%) under-5 children had normal weight-for-height, 889 (86.6%) had normal weight-for-age, and 850 (82.8%) had normal height-for-age Z-scores (Table 1).

**Table 1:** Socio-demographic and Anthropometric characteristics of Under-5 children and Caregivers in Urban slums of Ibadan, 2021

Characteristics	Total (N = 1027) n (%)
**Caregiver’s age (Years)**	
≤ 27	305 (29.7)
28–35	463 (45.1)
> 35	259 (25.2)
Mean age (± SD) years	31.7 ± 7.47
**Caregiver’s sex**	
Male	25 (2.4)
Female	1002 (97.6)
**Caregiver’s highest level of education**	
No formal	43 (4.2)
Primary	188 (18.3)
Secondary	753 (73.3)
Tertiary/Postgraduate	43 (4.2)
**Caregiver’s religion**	
Christianity	210 (20.4)
Islam	817 (79.6)
**Caregiver’s employment status**	
Unemployed	132 (12.9)
Employed	895 (87.1)
**Household wealth index**	
First (Poorest)	205 (20.0)
Second (Poorer)	205 (20.0)
Third (Average)	205 (20.0)
Fourth (Richer)	206 (20.0)
Fifth (Richest)	206 (20.0)
**Community type**	
Rural	501 (48.8)
Urban	526 (51.2)
**Age of children (months)**	
≤ 24	347 (33.8)
> 24–46	335 (32.6)
> 46	345 (33.6)
Mean age (± SD) months	34.49 ± 15.8
**Sex of children**	
Male	496 (48.3)
Female	530 (51.7)
**Weight-for-Height**	
Severe wasting	31(3.0)
Wasting	99 (9.6)
Normal	765 (74.5)
Overweight	99 (9.6)
Obese	33 (3.2)
**Weight-for-Age**	
Severely underweight	19 (1.9)
Underweight	119 (11.6)
Normal	889 (86.6)
**Height-for-Age**	
Severe stunting	44 (4.3)
Stunting	133 (13)
Normal	850 (82.8)

**Fig. 1:**
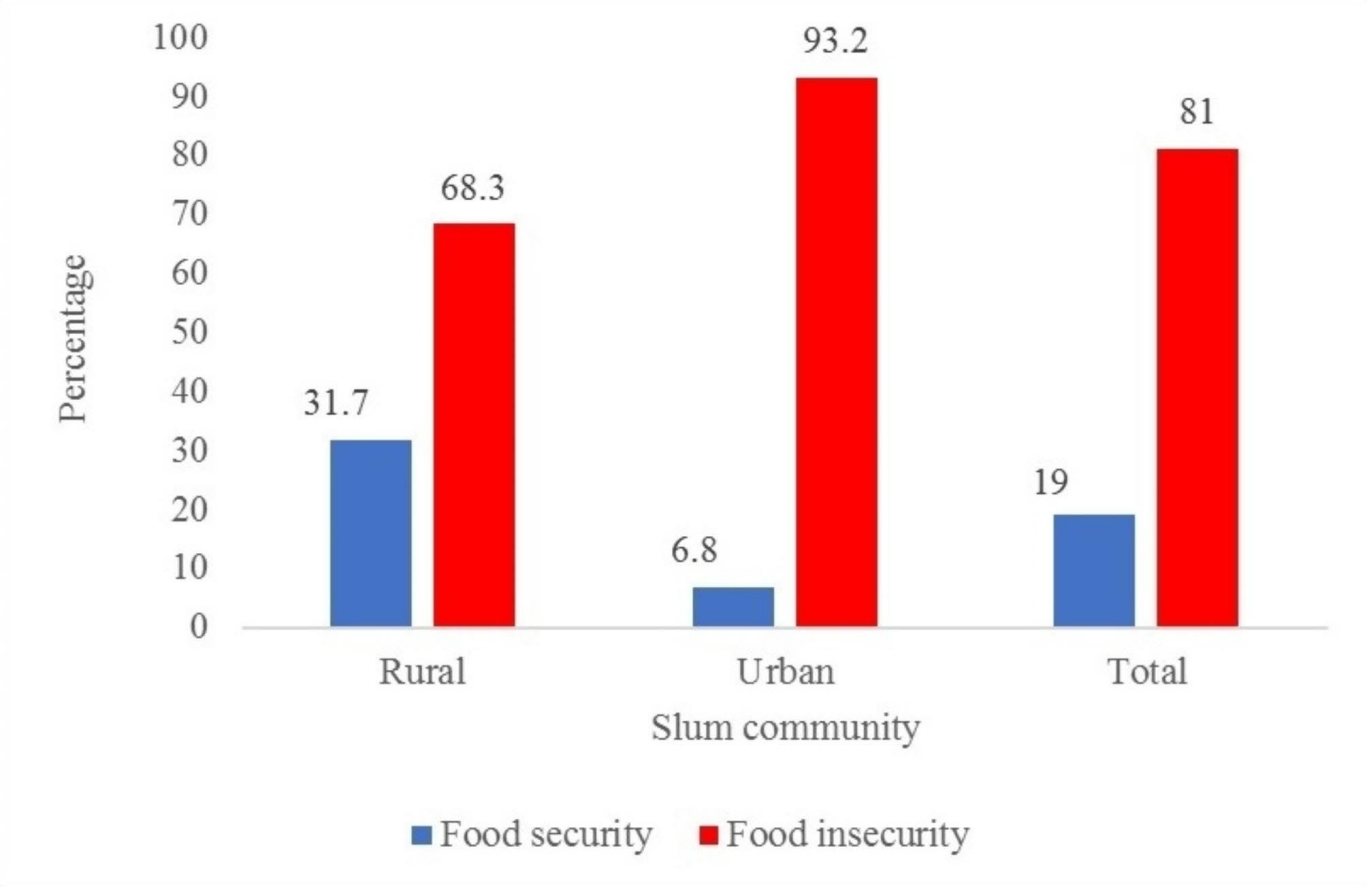
Prevalence of food insecurity among rural and urban slum households with under-5 children in Ibadan city, Nigeria

From Figs. 1 and 832 (81.0%) households in slum communities with under-5 children had food insecurity; 342 (68.3%) in rural slums and 490 (93.2%) in urban slums.

Table 2 describes the factors associated with food insecurity in households with under-5 children located within urban and rural slums in Ibadan. Overall, 87 (65.9%) households with under-5 children whose caregivers were unemployed had food insecurity compared to 745 (83.2%) households with employed caregivers (Chi-square = 22.463, p = < 0.001). Also, 342 (68.3%) households in rural slums had food insecurity compared to 490 (93.2%) in urban slums (Chi-square = 103.364, p = < 0.001). Compared to 708 (79.6%) households that had under-5 children with normal weight, 124 (89.9%) households that had under-5 children that were underweight or severely underweight were food insecure (Chi-square = 8.103, p = 0.004).

**Table 2:** Factors associated with household food insecurity in households with Under-5 children in urban and rural slums in Ibadan, Oyo State

Socio-demographic characteristics	Food Insecure N = 832 n (%)	Food Secure N = 195 n (%)	χ2	p-value
**Caregiver’s age (Years)**				
≤ 27	231 (75.7)	74 (24.3)	7.854	0.020
28–35	385 (83.2)	78 (16.8)		
> 35	216 (83.4)	43 (16.6)		
**Caregiver’s sex**				
Male	18 (72.0)	7 (18.0)	1.353	0.245
Female	814 (81.2)	188 (18.8)		
**Caregiver’s highest level of education**				
No formal	38 (88.4)	5 (11.6)	6.324	0.097
Primary	162 (86.2)	26 (13.8)		
Secondary	599 (79.5)	154 (20.5)		
Tertiary/Postgraduate	33 (76.7)	10 (23.3)		
**Caregiver’s religion**				
Christianity	195 (92.9)	15 (7.1)	24.076	< 0.001
Islam	637 (78.0)	180 (22.0)		
**Caregiver’s employment status**				
Unemployed	87 (65.9)	45 (34.1)	22.463	< 0.001
Employed	745 (83.2)	150 (16.8)		
**Wealth index**				
First (Poorest)	193 (94.1)	12 (5.9)	51.625	< 0.001
Second (Poorer)	178 (86.8)	27 (13.2)		
Third (Average)	165 (80.5)	40 (19.5)		
Fourth (Richer)	153 (74.3)	53 (25.7)		
Fifth (Richest)	143 (69.4)	63 (30.6)		
**Community type**				
Rural	342 (68.3)	159 (31.7)	103.364	< 0.001
Urban	490 (93.2)	36 (6.8)		
**Age of children (Months)**				
≤ 24	283 (81.6)	64 (18.4)	0.333	0.847
> 24–46	268 (80.0)	67 (20.0)		
> 46	281 (81.4)	64 (18.6)		
**Sex of children**				
Male	409 (82.3)	88 (17.7)	1.028	0.311
Female	423 (79.8)	109 (21.2)		
**Weight-for-Height**				
Severe wasting	30 (96.8)	1 (3.2)	8.369	0.079
Wasting	82 (82.8)	17 (17.2)		
Normal	615 (80.4)	150 (19.6)		
Overweight	82 (82.8)	17 (17.2)		
Obese	23 (69.7)	10 (30.3)		
**Weight-for-Age**				
Underweight/ Severely underweight	124 (89.9)	14 (10.1)	8.103	0.004
Normal	708 (79.6)	181 (20.4)		
**Height-for-Age**				
Severe stunting	38 (86.4)	6 (13.6)	0.866	0.649
Stunting	107 (80.5)	26 (19.5)		
Normal	687 (80.8)	163 (19.2)		

From model 1, under-5 children living in urban slums had six times higher odds of experiencing household food insecurity compared to those in rural slums (AOR = 6.485, 95%CI = 3.826–10.992, p = < 0.001). From model 2, under-5 children living in urban slums had seven times higher odds of experiencing household food insecurity compared to those in rural slums (AOR = 6.859, 95%CI = 4.524–10.509, p = < 0.001). Compared to obese under-5 children, those with severe wasting had 15 times higher odds of experiencing household food insecurity (AOR = 14.521, 95%CI = 1.610–130. 962, p = < 0.05), and overweight childrenhad three times higher odds of experiencing household food insecurity (AOR = 3.486, 95%CI = 1.250–9.724, p = < 0.05) (Table 3).

**Table 3:** Predictors of food insecurity in households with under-5 children in rural and urban slums in Ibadan city, Nigeria

Characteristics	Unadjusted logistic regression		Adjusted logistic regression	
	Odds Ratio	95% CI	Odds Ratio	95%CI
**Caregiver’s age (Years)**				
≤ 27	**1**		**1**	
28–35	1.590	1.062–2.383**	1.613	1.084–2.399**
> 35	1.305	0.799–2.131	1.360	0.852–2.171
**Caregiver’s sex**				
Male	0.849	0.311–2.338		
Female	**1**			
**Caregiver’s highest level of education**				
No formal	6.825	1.868–24.939***		
Primary	4.533	1.728–11.887***		
Secondary	2.234	0.952–5.241*		
Tertiary/Postgraduate	**1**			
**Caregiver’s religion**				
Christianity	0.992	0.492–2.001		
Islam	**1**			
**Caregiver’s employment status**				
Unemployed	**1**			
Employed	1.432	0.900–2.277		
**Household wealth index**				
First (Poorest)	4.634	2.285–9.395****	4.565	2.289–9.102****
Second (Poorer)	1.927	1.102–3.367**	1.986	1.154–3.419**
Third (Average)	1.546	0.929–2.572*	1.615	0.987–2.643*
Fourth (Richer)	1.214	0.738–1.997	1.295	0.811–2.069
Fifth (Richest)	1		**1**	
**Community type**				
Rural	**1**		**1**	
Urban	6.485	3.826–10.992****	6.895	4.524–10.509****
**Age of children (months)**				
≤ 24	1			
> 24–46	1.199	0.749–1.917		
> 46	1.024	0.660–1.588		
**Sex of children**				
Male	1.154	0.812–1.638		
Female	**1**			
**Weight-for-Height**				
Severe wasting	8.929	0.920–86.671*	14.521	1.610–130.962**
Wasting	1.535	0.497–4.473	2.096	0.746–5.892
Normal	2.306	0.922–5.766*	2.318	0.964–5.574**
Overweight	3.389	1.191–9.643**	3.486	1.250–9.724**
Obese	**1**		**1**	
**Weight-for-Age**				
Underweight/Severely unweight	2.360	1.112–5.010		
Normal	**1**			
**Height-for-Age**				
Severe stunting	1.391	0.503–3.844		
Stunting	0.932	0.537–1.618		
Normal	1			

*p < 0.1, **p < 0.05, ***p < 0.01, ****p < 0.001

## Discussion

Food insecurity was higher in the urban compared to the rural slum (93.2% versus 68.3%). The communal culture unique to the rural slum dwellers allows for food sharing practices among families. This may explain the lesser level of food insecurity amongst them as reported by this study. Findings revealed an overall prevalence of food insecurity of 81%. This high level of household food insecurity mirrors the high proportion observed in Ibadan (81%), Southern Nigeria (70%) and Malaysia (83%) but contrasts the findings of Roberts et al. (66.2%) [12, 19, 20]. Living in slums predisposes under-5 children to household food insecurity [21]. In a cross-sectional study conducted in East and West Gojjam zones of Amhara Region, Ethiopia, it was found that regional differences exist in food insecurity, with the eastern zone having a higher prevalence than the western zone [22].

Findings from this study revealed that households with under-5 children in urban slums are more likely to have food insecurity compared to those in rural slums. The higher prevalence of food insecurity in urban slums compared to rural slums observed in our study aligns with previous research conducted in various countries across Africa. A study conducted in Kenya found that households in urban slums were more susceptible to food insecurity due to limited access to land, low-income levels, and high food prices [11]. Similarly, research conducted in South Africa by Naicker and colleagues [23] highlighted that urban slum dwellers face significant challenges such as unemployment, inadequate infrastructure, and limited social support, which contribute to higher rates of food insecurity.

Beyond Africa, studies from different parts of the globe have also reported similar findings. A study conducted in India [24] revealed that households in urban slums face higher food insecurity compared to rural areas, attributed to rapid urbanization, overcrowding, and limited employment opportunities. Moreover, another study found that urban slums were more vulnerable to food insecurity due to socioeconomic disparities, limited access to quality healthcare, and inadequate public services [25]. Several underlying factors may explain the higher prevalence of food insecurity in urban slums compared to rural slums in Nigeria. First, rapid urbanization has led to increased population density in urban areas, resulting in limited access to land for agriculture. This restriction on agricultural activities reduces the capacity of urban households to produce their own food, making them more reliant on purchasing food from markets. Additionally, urban slum dwellers often face higher costs of living, including housing, transportation, and healthcare expenses, which further strains their ability to afford an adequate and diverse diet.

Another important factor contributing to food insecurity in urban slums is the limited availability of employment opportunities, particularly for low-skilled individuals [11]. The limited availability of stable and well-paying jobs exacerbate the economic vulnerability of many households in urban slums, making it difficult for them to afford nutritious food consistently. Furthermore, inadequate social support systems in urban slums, such as limited access to social welfare programs, can make households more vulnerable to food insecurity during times of economic shocks or crises.

The findings indicate that households in the poorest and poorer wealth quintiles are more likely to experience food insecurity compared to those in richer quintiles. These results are consistent with previous research conducted in other countries in Africa and globally that highlighted the pervasive nature of food insecurity among disadvantaged populations. In Kenya, Kimani-Murage and colleagues [11] found that households in the poorest wealth quintile were significantly more likely to experience food insecurity compared to wealthier households. The study attributed these disparities to limited financial resources, inadequate access to productive assets, and a lack of social safety nets among the poorest persons. Studies from other regions of the world also support the association between household wealth and food insecurity [26, 27]. These studies emphasize the importance of targeted interventions that address both income inequality and access to nutritious food in reducing household food insecurity.

Our findings demonstrate that weight-for-height is a significant predictor of household food insecurity. The results align with previous research that shows a strong association between weight-for-height and food insecurity. Weight-for-height, also known as the body mass index, has been widely used as a measure of nutritional status and is considered a reliable indicator of acute malnutrition among children and adults. Some studies have reported that househlds grappling with food insecurity are at a higher risk of raising children with poor weight-for-height measurement [28, 29]. Weight-for-height as a predictor of food insecurity can also be attributed to the complex interplay of social, economic, and environmental factors [28]. Low socioeconomic status, unemployment, and poverty are well-established determinants of food insecurity [28]. Individuals with limited financial resources may prioritize other essential needs over purchasing nutritious food, leading to compromised dietary quality and subsequent negative impact on weight-for-height status.

The strength of this research are as follows: Firstly, the study utilized a large sample size, thus the results reported are generalizable for the slum population in Ibadan. Secondly, validated questionnaires were used for data collection.

Due to the cross-sectional nature of this study, we could not investigate causal relationships between household food insecurity and the characteristics of children and their caregivers. Food insecurity was self-reported; therefore, the risk of social desirability bias was high. Since anthropometry measurements are prone to measurement bias, some misclassification of children’s nutritional status might not be ruled out. However, we standardized anthropometric instruments, and conducted intensive training sessions with the the research assistants. We also closely supervised the data collection exercise to minimize anthropometric measurement errors and interviewer bias.

## Conclusion

Household food insecurity was more prevalent in urban slums compared to rural slums. This study found that households with under-5 children that are severely wasted, overweight and underweight are likely to be experiencing food insecurity. In other words, malnutrition served as a predictor of household food insecurity in this study. The findings of this study have significant implications for public health interventions and policy efforts aimed at addressing food insecurity. The government, through her relevant ministries in collaboration with partners should implement entrepreneurial trainings to improve the financial capacity of the slum dwellers. This will improve their purchasing power and access to diverse diets.

Information Education and Communication materials should be on permanent display at all immunization centres to avail mothers and/or caregivers with the right information about preventing malnutrition. Strengthening of the school health program would also go a long way ensuring that children who are not doing well will be picked up on time and attended to accordingly. By recognizing weight-for-height as a significant predictor of household food insecurity, healthcare providers, community organizations, and policymakers can utilize this information to identify at-risk populations and develop targeted interventions. Screening individuals or households for weight-for-height measurements can serve as an effective tool for early identification and intervention, allowing for timely provision of appropriate support and resources.

## Data Availability

The datasets used and/or analysed during the current study are available from the corresponding author on reasonable request.
